# Visualization of the protein–protein interactions of hormone receptors in hormone-dependent cancer research

**DOI:** 10.1530/EO-22-0059

**Published:** 2022-10-03

**Authors:** Erina Iwabuchi, Yasuhiro Miki, Takashi Suzuki, Hironobu Sasano

**Affiliations:** 1 Department of Pathology, Tohoku University Graduate School of Medicine, Sendai, Japan; 2 Department of Pathology and Histotechnology, Tohoku University Graduate School of Medicine, Sendai, Japan; 3 Department of Disaster Obstetrics and Gynecology, International Research Institute of Disaster Science (IRIDes), Tohoku University, Sendai, Japan

**Keywords:** protein–protein interactions, steroid hormone receptor, breast cancer, prostate cancer, proximity ligation assay

## Abstract

In hormone-dependent cancers, the activation of hormone receptors promotes the progression of cancer cells. Many proteins exert their functions through protein–protein interactions (PPIs). Moreover, in such cancers, hormone–hormone receptor binding, receptor dimerization, and cofactor mobilization PPIs occur primarily in hormone receptors, including estrogen, progesterone, glucocorticoid, androgen, and mineralocorticoid receptors. The visualization of hormone signaling has been primarily reported by immunohistochemistry using specific antibodies; however, the visualization of PPIs is expected to improve our understanding of hormone signaling and disease pathogenesis. Visualization techniques for PPIs include Förster resonance energy transfer (FRET) and bimolecular fluorescence complementation analysis; however, these techniques require the insertion of probes in the cells for PPI detection. Proximity ligation assay (PLA) is a method that could be used for both formalin-fixed paraffin-embedded (FFPE) tissue as well as immunostaining. It can also visualize hormone receptor localization and post-translational modifications of hormone receptors. This review summarizes the results of recent studies on visualization techniques for PPIs with hormone receptors; these techniques include FRET and PLA. In addition, super-resolution microscopy has been recently reported to be applicable to their visualization in both FFPE tissues and living cells. Super-resolution microscopy in conjunction with PLA and FRET could also contribute to the visualization of PPIs and subsequently provide a better understanding of the pathogenesis of hormone-dependent cancers in the future.

## Introduction

**Figure 1:**
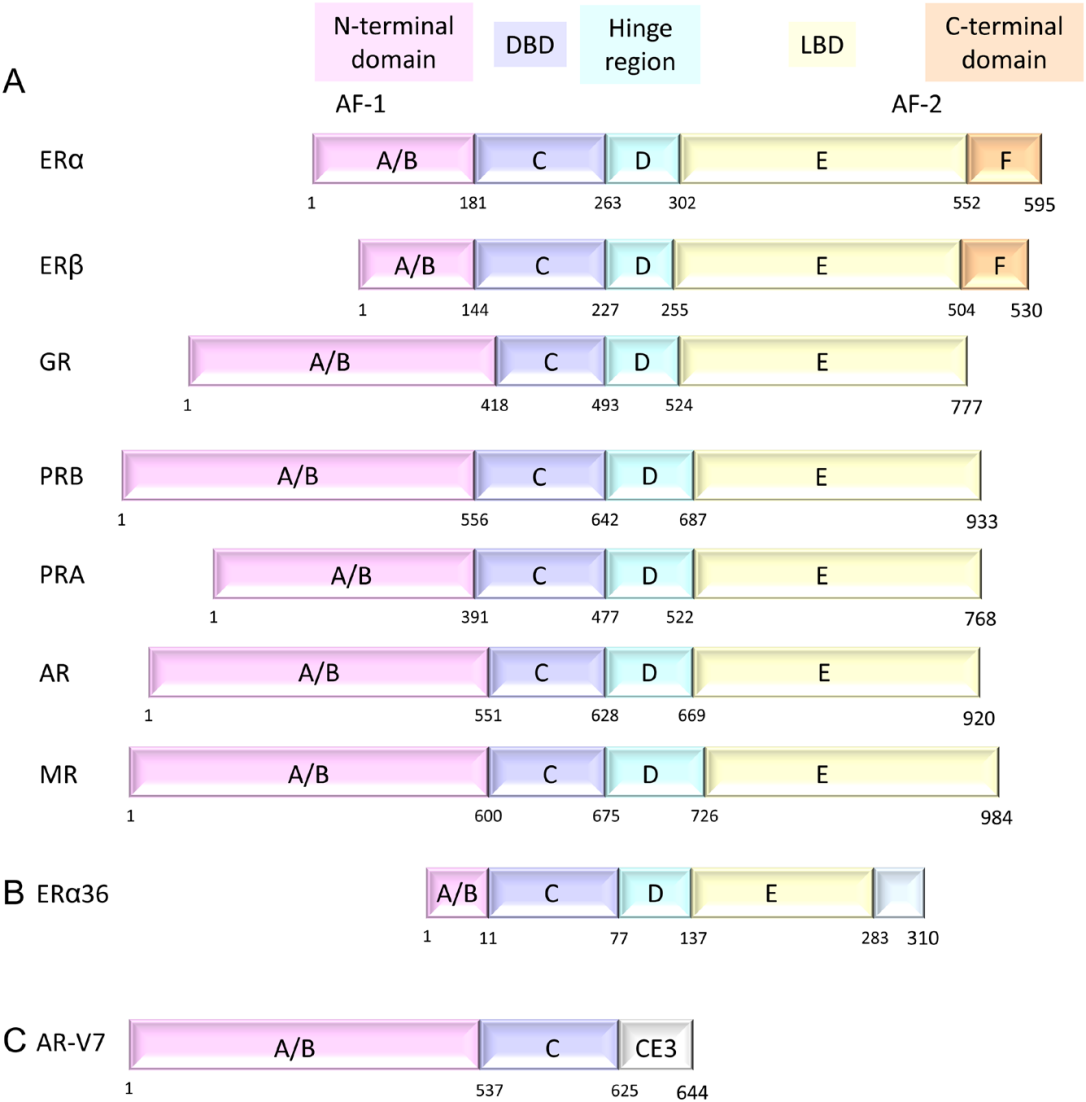
Summary of the steroid hormone receptor family and visualization of hormone receptor dimerization. (A) Hormone receptors include estrogen receptor (ER), progesterone receptor (PR), glucocorticoid receptor (GR), androgen receptor (AR), and mineralocorticoid receptor (MR). The hormone receptor is composed of the N-terminal domain (A/B), a flexible hinge region (D), and a C-terminal ligand-binding domain (LBD, E). ER, which has two isoforms, ERα and ERβ, is unique in that it contains an additional C-terminal F domain. PR has two isoforms PRA and PRB. The number of amino acid sequences is shown on the right. They have the two transcriptional activation functions through the AF-1, located in the N-terminal domain, and through the AF-2, located in the ligand-binding domain. This is important for forming the coregulator binding site as well as mediating direct interactions between the N-terminal and ligand-binding domains that form and function as homodimers or heterodimers. (B) ER splice variant ERα36 retains the DNA-binding domain but lacks both transactivation domains, AF-1 and AF-2, and differs with the unique C-terminal domain. (C) AR splice variants AR-V7 that contain the N-terminal domain and DNA-binding domain and lacks the LBD; however, the unique C-terminal domain, alternative splicing of the gene truncates the protein leaving only cryptic exon 3 (CE3) at the C-terminus.

Cancer is the most commonly diagnosed life-threatening disease, with an increasing number of cancer-related deaths worldwide each year (Sung *et al.* 2021). Among various cancer types, hormone-dependent cancers are characterized by their dependence on steroid hormones, which contribute to cancer progression, and these include breast, endometrial, ovarian, prostate, and testicular cancers. The biological effects of steroid hormones are mediated by the associated receptors, which function as transcription factors in steroid-responsive cells (Soica *et al.* 2020). Hormone receptors include estrogen, progesterone, glucocorticoid, androgen, and mineralocorticoid receptors (Fig. 1A). The hormone receptors generally comprise three different primary functional domains, i.e. the N-terminal, DNA-binding, and ligand-binding domains (Mangelsdorf *et al.* 1995, Hovland *et al.* 1998, Kumar *et al.* 2011, Jimenez-Panizo *et al.* 2019). These domains have two transcriptional activation functions: through the AF-1, which is located in the N-terminal domain, and through the AF-2, which is located in the ligand-binding domain. These two functions are important for forming the coregulator binding site as well as mediating direct interactions between the N-terminal and ligand-binding domains, which function as homodimers or heterodimers following their binding (Mangelsdorf *et al.* 1995, Hovland *et al.* 1998, Kumar *et al.* 2011, Jimenez-Panizo *et al.* 2019). Moreover, ligands binding to these steroid hormone receptors cause cascading events (i.e. structural conformational changes, partial release of chaperone proteins, and dimerization), and eventually the recruitment of cofactor molecules. These cofactors bind to response elements in the promoter region of the specific genes and cause the activation and/or repression of gene transcription (Edman *et al.* 2015). The expression of these cofactors is generally considered an important factor for the determination of the tumor response to steroid hormone stimulation (Soica *et al.* 2020). Therefore, it is believed that hormone receptors play a key role in cancer development as they promote the growth of these tumors.

## Protein–protein interactions are important in understanding protein function

Overall, >80% of proteins function by forming complexes rather than remaining as a single entity, and the functions of many proteins (signal transduction, transport, and metabolism) depend on structural changes and responses to interactions with other proteins (Rao *et al.* 2014). Therefore, elucidating protein–protein interactions (PPIs) of hormone receptors is expected to lead not only to a better understanding of hormone signaling and the pathogenesis or development of hormone-dependent cancers but also to the development of therapeutic resistance (Miki *et al.* 2018). Steroid hormone receptors are usually visualized using immunohistochemistry in formalin-fixed paraffin-embedded (FFPE) tissues. For breast cancers, immunohistochemistry of steroid hormone receptors has been used in clinical practice for the selection of specific therapies (Allred 2010). Therefore, it is reasonable to assume that the visualization of PPIs will help provide additional information on the pathophysiology of hormone-dependent cancers. In this review, we summarize the results of recently published studies on the visualization of PPIs in hormone-dependent cancers, including breast and prostate cancers.

PPIs visualization methods include Förster resonance energy transfer (FRET), proximity ligation assay (PLA), and bimolecular fluorescence complementation (BiFC) assay. FRET is based on the phenomenon that occurs when one fluorescent molecule’s (donor) fluorescence spectrum and another fluorescent molecule’s (acceptor) excitation spectrum overlap while the two molecules are in close proximity (<10 nm). When the dipole moments of both molecules are in an appropriate directional relationship, the excitation energy has a high probability of exciting the acceptor before the emission from the donor occurs (Muller *et al.* 2013). BiFC analysis visualizes PPIs by fusing two proteins with nonfluorescent fragments of a fluorescent protein, based on when the two proteins interact and the fluorescent protein reconstitutes (Kerppola 2006). FRET can be detected at a distance of approximately 1–10 nm, whereas BiFC can be detected at a distance of approximately 7 nm. Additionally, monitoring PPI visualization in living cells may be achieved using both FRET and BiFC analyses; however, FRET is distinguished by reversible interactions between its components, but BiFC is not.

**Figure 2:**
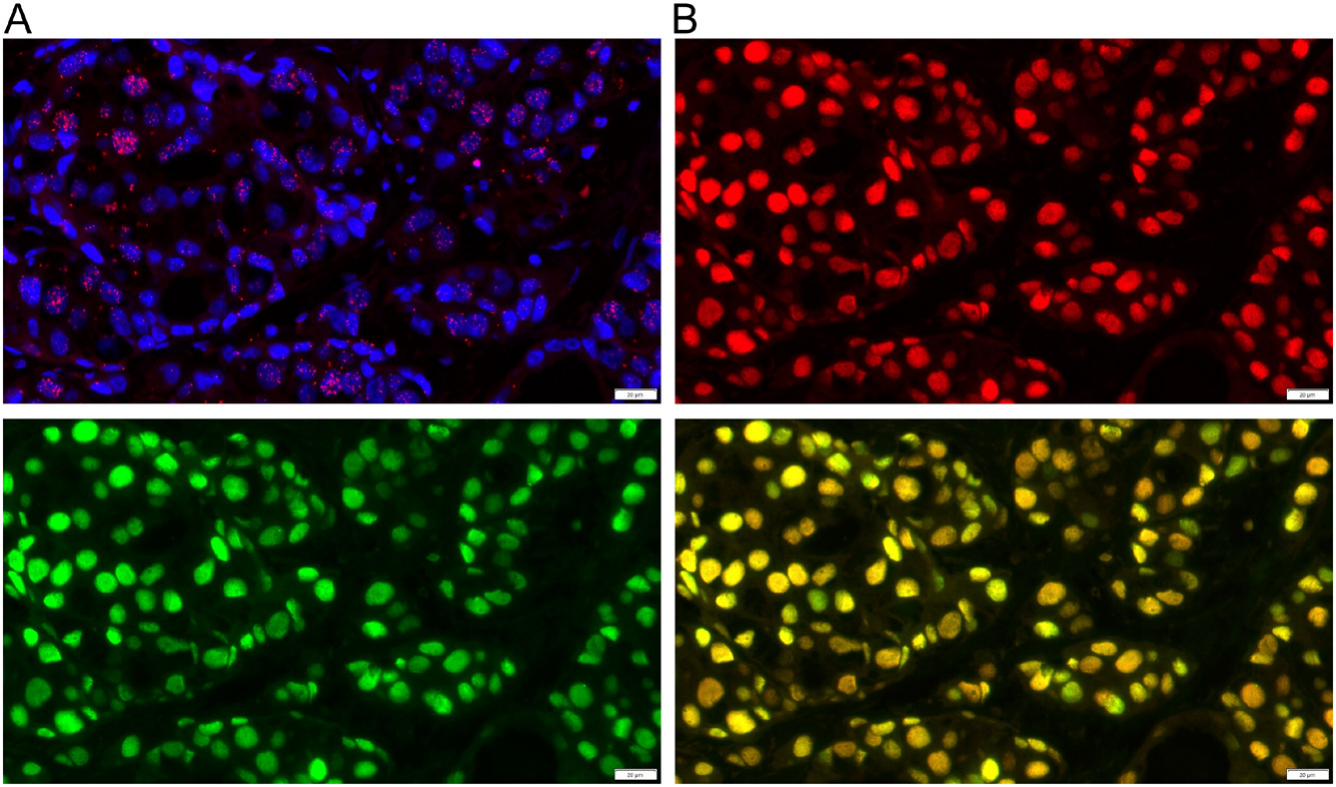
Visualization of ERα homodimers and ERα immunoreactivity in formalin-fixed paraffin-embedded breast cancer tissues. (A) ERα homodimers is indicated by red dots. Nucleus is shown in blue (DAPI). (B) ERα immunoreactivity is shown in green and red, respectively. Double positive cells are shown in yellow.

*In situ* PLA has been developed to visualize interacting proteins in fixed cells or tissues (Soderberg *et al.* 2006). This method is based on the use of oligonucleotide-conjugated antibodies, called PLA probes, each with a unique short DNA strand attached to and bound to the primary antibodies. When the PLA probes are located in close proximity (<40 nm), the DNA strands can interact through subsequent addition of two other circle-forming DNA oligonucleotides (Soderberg *et al.* 2006). After merging of the two added oligonucleotides by enzymatic ligation, they are amplified through rolling circle amplification and subsequently detected using a fluorescently labeled probe or horseradish peroxidase-based signals (Soderberg *et al.* 2006). Among these methods, the PLA method is highly versatile because it does not require gene transfer into the cells and can be detected by an antigen–antibody reaction using an antibody generally employed in immunohistochemistry. PLA can also be used in FFPE tissues. Therefore, the method is expected to have potential as a new pathological evaluation method (Iwabuchi *et al.* 2021*a*) (Fig. 2). PPIs of hormone receptors using the PLA have been reported not only in hormone-dependent cancers but also in other normal or nonpathological cells (Table 1).

**Table 1:** Summary of protein–protein interactions of hormone receptors using the proximity ligtion assay.

First author	Protein–protein interactions	Samples	Hormone-dependent cancers or healthy cells	Year
Zieba *et al.*	ERα/estradiol	Human tissues	Breast cancer	2010
Vernocchi *et al.*	GR/caveolin-1	MCF-7 cells	Breast cancer	2013
		U2-OS cells	Osteosarcoma	
Abot *et al.*	ERα/Src	MCF-7 cells	Breast cancer	2014
Jehle *et al.*	AR/Bag-1	LNCaP cells	Prostate cancer	2014
Iwabuchi *et al.*	ERα/ERα, ERα/ERβ	Human tissues	Breast cancer	2017
		MCF-7 cells, T-47D cells		
Sreenath *et al.*	AR/ETS-related gene	LNCaP-LnTE3 cells, VCaP cells	Prostate cancer	2017
Snell *et al.*	ERα/PRB	Human tissues	Breast cancer	2018
Grolez *et al.*	AR/transient receptor potential melastatin 8	LNCaP cells	Prostate cancer	2019
Auvin *et al.*	AR/proteasome, AR/SUMO1,	LNCaP cells	Prostate cancer	2019
	AR/SUMO2/3			
	AR/promyelocytic leukemia			
Pooley *et al.*	GR/MR	3617 cells	Mouse mammary	2019
Majumdar *et al.*	ERs/caveolin-1	Human primary prostate epithelial cells	Prostate epithelial cells	2019
	ERα/ERβ2			
Konan *et al.*	ERα36/PR	T-47D cells	Breast cancer	2020
Bahnassy *et al.*	AR/SUMO	MCF-7 cells	Breast cancer	2020
Lozovyy *et al.*	Progesterone receptor membrane component/progesterone	Human primary amnion epithelial cells, amnion mesenchymal stromal cells, chorion cells	Amnion epithelial cells, amnion mesenchymal stromal cells, chorion cells	2021
Dwyer *et al.*	PR/insulin receptor substrate-1	T-47D cells	Breast cancer	2021
Jehanno *et al.*	ERα/ERα, ERα/HSP70, ERα/SRC1,	MCF-7 cells	Breast cancer	2021
	ERα/SRC3, ERα/SMRT,			
	ERα/NCOR1, ERα/c-src,			
	ERα/p85PI3K			
Iwabuchi *et al.*	ERα/hnRNPK	MCF-7 cells	Breast cancer	2021
Mirzakhani *et al.*	AR/AKT	LNCaP cells	Prostate cancer	2022

AR, androgen receptor; ER, estrogen receptor; GR, gulcocorcicoid receptor; MR, mineralocorticoid receptor; PR, progesterone receptor; SUMO, small ubiquitin-like modifier.

## Binding of receptors to hormones

Steroid hormone receptors are transcription factors that function in the nucleus, depending on the ligand steroid hormone. The classic genomic pathway is generally characterized by regulating the transcriptional activity and gene expression of various target genes. In contrast, steroid hormones are known to act in the cytoplasm and cell membrane without influencing transcriptional regulation or new protein synthesis and are believed to correspond to the nongenomic pathway, leading to acute cellular responses (Wilkenfeld *et al.* 2018).

Visualization of the binding of this steroid hormone to the hormone receptor has also been reported using PLA. Zieba* et al*. reported that the binding of estradiol to estrogen receptor (ER)-α can be visualized in breast cancer tissue using *in situ* PLA. The results revealed that the differences between ERα-positive and ERα-negative tumors were highly significant for ER and ERα–estradiol complex detection and significant for estradiol detection (Zieba *et al.* 2010). In addition, Lozovyy* et al*. reported the visual evaluation of the binding between progesterone (P4) and progesterone receptor membrane component (PGRMC) in human primary amnion epithelial cells, amnion mesenchymal stromal cells, and chorion cells, thereby allowing the interaction between P4 and PGRMC in all fetal membrane cell types. P4–PGRMC complexes contribute to maintaining functional completeness of the membrane (Lozovyy *et al.* 2021).

## ER dimerization

Steroid hormone receptors form homodimers or heterodimers via the DNA-binding domain. ER has two isoforms, α and β, that form homodimers or heterodimers. In breast cancer cells, ERα promotes the growth of cancer cells, whereas ERβ suppresses the growth of these cells. Thus, heterodimers are thought to suppress the growth of breast cancer cells. In prostate cancer, ERβ was reported to be involved in the differentiation of prostatic epithelial cells and in anti-proliferative actions (Christoforou *et al.* 2014). Bai and Giguere reported the identification of ER dimeric proteins using FRET analysis. They demonstrated that the formation of ERα and ERβ homodimers and heterodimers occurs independently of 17β-estradiol (E2) in living cells (Bai & Giguere 2003). Using PLA, we previously reported the presence of ERα homodimers and ER heterodimers in breast cancer cells and tissues (Iwabuchi *et al.* 2017*b*) (Fig. 2). ERα homodimer in MCF-7 cells was increased gradually by E2 treatment after 15 and 45 min and decreased after 90 min. We then examined the association between the number of ERα homodimers and the expression of breast cancer biomarkers in 25 patients with breast cancer. ERα homodimer was significantly higher in high ERα or progesterone receptor (PR) expression cases than in low expression cases, and it was significantly lower in the cases associated with higher Ki67 expressions. In contrast, ER heterodimer was significantly higher in high ERα expression cases than in low expression cases, but there was no significant association with ERβ expressions (Iwabuchi *et al.* 2017*b*).

## PR dimerization

The PR isoform is generally expressed as two isoforms, PRA and PRB, which are transcribed from a single gene (*PGR*) (Conneely *et al.* 2003). PRA functions in the progesterone-dependent reproductive response required for female fertility, and PRB functions to elicit normal proliferative and differentiation responses in the mammary gland (Conneely *et al.* 2003). Aberrant ratios of PR isoforms have been reported in endometrial cancers, and overexpression of PRB is associated with highly malignant forms of endometrial, cervical, and ovarian cancers (Richer *et al.* 2002). However, the functions of PR dimerization patterns have remained unknown. Visualization of PR homodimers and heterodimers have been reported as showing increased FRET signal upon progestin ORG2058 treatment in human osteosarcoma cell line U-2 OS cells transfected with CFP-PRA, YFP-PRA, CFP-PRB, and YFP-PRB, respectively (Arnett-Mansfield *et al.* 2007). Both PR homodimer and heterodimer were induced in ORG2058-treated cells. The FRET signal of PRA homodimer increased three-fold in whole nuclei and four-fold in PRA foci of ORG2058-treated cells compared with control. The FRET signal of PRB homodimer increased four-fold in whole nuclei and six-fold in PRB foci in ORG2058-treated compared with the controls. The FRET signal of PRA/PRB heterodimer in ORG2058-treated whole nuclei was increased by two- to three-fold and three- to four-fold in foci compared with the control cells (Arnett-Mansfield *et al.* 2007).

With the use of immunoprecipitation, T-47D, ER, and PRB complexes in breast cancer cells increased in the presence of both ER and PR agonist ligands (Singhal *et al.* 2016). ER and PRB form heterodimers in the presence of their cognate hormones and this activity may promote better disease outcomes (Mohammed *et al.* 2015). In addition, progesterone inhibited estrogen-mediated growth of ERα-positive breast cancer cells and increased anti-proliferative effects when coupled with an ERα antagonist (Mohammed *et al.* 2015). Moreover, Snell* et al*. detected ER and PRB interactions in 229 patients with ER-positive and HER2-negative breast cancer tissues using PLA. ER and PRB interaction frequency was an independent predictive factor for relapse, whereas PR expression was not. Furthermore, low frequency of ER and PRB interactions was predictive of relapse on adjuvant aromatase inhibitors (Snell *et al.* 2018).

ERα36 is encoded by the ESR1 locus and retains the DNA-binding domain but lacks both transactivation domains, AF-1 and AF-2, and differs with the unique C-terminal domain (Wang *et al.* 2005, Porras *et al.* 2021) (Fig. 1B). ERα36 was reported to activate ERK phosphorylation and enhance cyclin D1/CDK4 expressions, leading to an increased cell cycle progression (Konan *et al.* 2020). It has been shown that ERα36 interacts with PR in the nucleus of T-47D cells. ERα36 regulated PR signaling and inhibited its transcriptional activity; moreover, it inhibited progesterone-induced anti-proliferative and anti-migratory effects (Konan *et al.* 2020).

## Androgen receptor dimerization

In the case of androgen receptor (AR), activation of AR by androgen is known to be involved in the progression of prostate cancer, and antiandrogen drugs that block the binding are used for treatment. AR agonists (testosterone, dihydrotesterone, and R1881) induced intramolecular AR N-terminal domain and AR ligand-binding domain interactions in the nucleus and cytoplasm (Nadal *et al.* 2017). In contrast, no FRET effect was observed in the presence of the AR antagonists – enzalutamide, bicalutamide, or hydroxyflutamide (Nadal *et al.* 2017). In addition, it has been reported that AR and AR splice variant 7 (AR-V7) form homodimers and heterodimers, respectively (Xu *et al.* 2015), with the AR variant AR-V7, which contains the N-terminal domain and DNA-binding domain and differ with the unique C-terminal domain, alternative splicing of the gene truncates the protein, leaving only cryptic exon 3 at the C-terminus (Dehm & Tindall 2011, Vickman *et al.* 2020) (Fig. 1C). AR-V7 is generally higher in castration-resistant tumors than in androgen-dependent tumors. AR-V7 was reported to enhance growth of androgen-dependent xenografts in castrated mice; hence, it has been reported to be important for the progression of castration-resistant prostate cancer (Guo *et al.* 2009). However, AR-V7 homodimers and AR and AR-V7 heterodimers functions and clinical significance have not been fully investigated and need to be clarified.

## Glucocorticoid receptor dimerization

The human glucocorticoid receptor (GR) gene expresses two splicing isoforms α and β (Noureddine *et al.* 2021). GR has also been reported to be involved in cancer progression in breast cancer, and its potential as a therapeutic target is being investigated (Noureddine *et al.* 2021). GRα was reported to be responsible for most glucocorticoid-mediated transcriptional activities, but GRβ was not able to bind to endogenous glucocorticoids or activate glucocorticoid-responsive reporter and endogenous genes (Oakley *et al.* 1999). GRβ was reported to have effects on GRα-induced transcriptional activity. In addition, GRα/GRβ heterodimers were reported to be transcriptionally inactive or less active than a GRα homodimer, so that heterodimer is thought to act to suppress the function of GRα (Oakley *et al.* 1996). Therefore, it has been suggested that GR is involved in transcription of different factors in monomers and dimers, and it is extremely important to know in which state it exists (Timmermans *et al.* 2022). In addition, it has been reported that GR forms a heterodimer with MR and that GR/MR heterodimers have a gene regulatory role distinct from GR or MR alone, but its function has not been clarified, and future studies using visualization of complexes are needed (Pooley *et al.* 2020). Moreover, several studies that suggested the formation of GR/AR heterodimers have also been reported, and we also report on the interaction of AR and GR in triple-negative breast cancer (Kanai *et al.* 2020). Our results indicated that interaction of GR and AR might be involved in the suppression of GR-induced migration by AR signaling (Kanai *et al.* 2020).

## Hormone receptors and cofactor complexes

Transcriptional activation of target genes by nuclear receptors is regulated by cofactors (coactivators that promote transcription and corepressors that suppress transcription). The binding of the agonist to the ligand-binding site makes it easier to bind to the coactivator, and the formed transcription factor complex promotes the action of the basic transcription factor complex including RNA polymerase II. Conversely, when an antagonist binds, it recruits a corepressor and suppresses transcriptional activation.

There are also many reports on the visualization of cofactor-receptor binding. Bai and Giguere reported ER dimerization as well as interaction between ERα and coactivator. The SRC family consists of SRC-1/NcoA1, SRC-2/NcoA2, and SRC-3/NcoA3 (Bai & Giguere 2003). The interactions between SRCs and nuclear receptors are mediated through receptor-interacting domains (RIDs) and receptor ligand-binding domains (Bai & Giguere 2003). They showed that interaction between ERα and RIDs of SRC-1, SRC-2, and SRC-3 with or without E2 using FRET (Bai & Giguere 2003). ERα and SRC interactions were detected using PLA. Abot* et al*. attempted to visualize the formation of ERα and SRC interactions by Estriol (E4). They demonstrated that E4 promoted ERα and SRC interactions less efficiently than E2 but induces similar ERE-dependent transcriptional activity in MCF-7 (Abot *et al.* 2014). Jehanno* et al*. reported changes in the interaction between ERα and SMRT (coregulator functions as a dual coactivator and corepressor) and NCOR1 corepressor, and the interaction between ERα and kinases, such as c-src and PI3K, using the PLA method. The results showed that activation and nuclear accumulation of MRTFA in ERα-positive breast cancer cells shifts ERα nuclear/genomic action to extranuclear/nongenomic action (Jehanno *et al.* 2021). They revealed the interactions between endogenous ERα and IFI27/ISG12 in the cytoplasm, nucleus, and perinuclear region in breast cancer cell MCF-7, T47D, and ZR-75-1 cells. IFI27/ISG12 overexpression was shown to suppress the estradiol-dependent proliferation and tamoxifen-induced apoptosis in breast cancer cells. They suggested that IFI27/ISG12 is an important factor in regulating ERα activity in breast cancer cells and a potential target of future strategies to control the growth and proliferation of ERα-positive breast cancer (Cervantes-Badillo *et al.* 2020).

The cochaperone Bag-1L, Bag-1 isoform is known to be one of the factors regulating the activity of AR (Jehle *et al.* 2014). Jehle *et al*. showed the interaction between AR and Bag-1 in the LNCaP cells in the absence and presence of dihydrotestosterone (DHT) (Jehle *et al.* 2014). The mutation of the GARRPR motif of Bag-1L leads to the inhibition of the AR and Bag-1L interaction, and they indicated that binding of Bag-1L to the AR through the GARRPR motif contributes to the suppression of a part of AR-regulated genes (Jehle *et al.* 2014). SKIP also interacts with AR in the nucleus and promotes AR-dependent transcriptional activation, suggesting that SKIP functions as a cofactor for AR. Abankwa* et al*. show SKIP increased DHT-induced N-terminal/C-terminal AR interaction and enhanced AF-1 transactivation. FRET analysis suggested a direct AR and SKIP interaction in the nucleus upon translocation, and SKIP interacts with AR in the nucleus and enhances AR-dependent transactivation and AR dimerization supporting a role for SKIP as an AR cofactor (Abankwa *et al.* 2013).

## Protein–protein interactions between hormone receptor and several other proteins

PR has been reported to be regulated by the interaction between ERα and Sp1 (Schultz *et al.* 2003). Kim *et al*. have reported that ERα and Sp1 proteins fused to cyan fluorescent protein or yellow fluorescent protein were transfected into MCF-7 cells and that a FRET signal was induced by ERα agonists/antagonists (Kim *et al.* 2005).

In a study using prostate stem/progenitor cells to evaluate the subcellular localization of ERs, we visualized the PPIs of ERs with caveolin-1, a lipid raft membrane marker, and reported that ERs are on the cell membrane. As described earlier, the PLA method is used not only to clarify the intracellular localization of PPIs but also to clarify the intracellular localization of a single protein (Majumdar *et al.* 2019). In addition, prostate cancer stem-like cells express only ERβ, and E2 treatment activated the MAPK pathway via ERβ. They showed membrane-associated ERα and ERβ differentially engaged downstream signaling pathways in normal and oncogenic prostate stem/progenitor cells. These signaling pathways could affect normal prostate stem/progenitor cell homeostasis and provide new therapeutic sites for targeting prostate cancer stem cells (Majumdar *et al.* 2019).

Heterogeneous nuclear ribonucleoprotein (hnRNPK) is a protein involved in chromatin remodeling, transcription, splicing, and translational processes, and our findings have shown that it functions as a binding protein for ERα. In addition, ERα and hnRNPK interacted directly and were involved in ER-mediated signaling pathways in breast cancer (Iwabuchi *et al.* 2021*b*). Furthermore, we reported that Fe65, a binding protein of amyloid precursor protein (APP), was translocated into the nucleus by phosphorylation of APP and was involved in promoting cancer cell progressions in breast cancer (Xu *et al.* 2022). Fe65 has also been known to bind to the ER, and it is required to clarify the impact of these ER-binding proteins on estrogen signaling and their relationship to the therapeutic response of patients to endocrine therapy.

The insulin-like growth factor (IGF)/insulin signaling pathway is involved in the cancer cell progression of various human malignancies. Through ligand binding, IGF1 receptorβ (IGF1Rβ), insulin receptor (IR), and IGF1Rβ/IR complex receptors recruit insulin receptor substrate (IRS) adaptor proteins and effect on downstream signal transduction (Byron *et al.* 2006). IRS-1 was the major known isoform and reported to promote tumor growth in breast cancer (Byron *et al.* 2006). Dwyer* et al*. demonstrated that E2 induced interaction between PRB and IRS-1 using PLA and their complexes contributed to promote growth of endocrine-resistant and stem-like breast cancer cells (Dwyer *et al.* 2021).

Visualization of AR interactions with other proteins has been reported variously in prostate cancer cells. They show that ETS-related gene (ERG), which has been well established in diverse human cancers, through its physical interaction with AR in prostate cancer LNCaP-LnTE3 cells using PLA, induces AR aggregation and endoplasmic reticulum stress. They further show that continued expression of ERG leads to evade cell death through activation of cell survival pathways (Sreenath *et al.* 2017). Furthermore, androgens were known to regulate transient receptor potential melastatin 8 (TRPM8) protein expression through AR activation in prostate cancer development, and TRPM8 activity was reported to suppress prostate cancer cell migration (Grolez *et al.* 2019). Grolez *et al.* demonstrated that endogenous TRPM8 interacted directly with the AR in prostate cancer LNCaP cells, but their interaction was reduced by treatment of testosterone using PLA. Their findings identified a nongenomic mechanism of the TRPM8 channel regulation by androgens (Grolez *et al.* 2019). In addition, Mirzakhani *et al.* reported that AR and AKT interaction and AKT phosphorylation were induced by supraphysiological androgen levels treatment and lead to cellular senescence (Mirzakhani *et al.* 2022).

GRs, which are similar to other steroid hormone receptors, exert their rapid nongenomic effects by several mechanisms including the activation of a membrane-bound glucocorticoid receptor (mGR) (Vernocchi *et al.* 2013). Vernocchi* et al*. reported that mGR localization is present in caveolae in human osteosarcoma U2-OS cells and MCF-7 cells by visualization of its interaction with caveolin-1 using PLA (Vernocchi *et al.* 2013).

PLA can also be applied to detect post-translational modifications using an antibody against the modification and an antibody against the target protein. For example, SUMOylated AR has also been reported in both breast and prostate cancer using PLA. SUMOylation is a post-translational modification that is involved in various cellular processes, such as nuclear trafficking, transcriptional regulation, apoptosis, protein stability, stress response, and cell cycle. SUMO proteins are three isoforms (SUMO-1, SUMO-2, and SUMO-3) that have been identified in humans. De-SUMOylation is potentially catalyzed by a family of SUMO-specific proteases (SENPs). Silencing of SENP1 has also been reported to suppress the expression of several AR target genes and to inhibit androgen-stimulated proliferation of LNCaP cells, thus the SUMOylation pathway is a potential therapeutic target for prostate cancer (Kaikkonen *et al.* 2009). Bahnassy* et al*. visualized the subcellular localization of SUMO and AR interactions in PLA to detect SUMOylated AR. Overexpression of SUMO3 in MCF-7 transferred the interaction between AR and SUMO from cytoplasm to nucleus (Bahnassy *et al.* 2020). Similarly, in prostate cancer, it was reported that both SUMO1 and AR interactions (in IRC117539, which is a new molecule that targets AR for proteasomal degradation, untreated and treated cells) and SUMO2/3 and AR interactions (in IRC117539 treated cells) were largely nuclear in LNCaP cells using PLA (Auvin *et al.* 2019).

PLA has also been used to reveal the localization of arginine methylation in cells. Poulard* et al*. have attempted to detect methylation of GR using a pan-methyl antibody that specifically recognizes symmetrical dimethylation (SDMA). They visualized the interaction between GR and SDMA in MCF-7. Furthermore, they demonstrated that arginine methyltransferase PRMT5 depletion significantly decreased GR and SDMA interaction as detected by PLA. PLA is expected to be used to detect not only methylation but also other post-translational modifications (Poulard *et al.* 2020).

## Super-resolution microscopy analysis

We previously reported the visualization of ER dimers using super-resolution microscopy, which is an optical technique with resolution exceeding the diffraction limit of conventional optical microscopy (Iwabuchi *et al.* 2017*a*). Conventional light microscopy revealed many proteins that appeared to be colocalized; this prevented the detailed analysis of their spatial relationships. For functional analysis of cells, the visualization of fine structures and spatial changes in intracellular organelles as well as the intracellular localization of proteins at the organelle level are required. In our previous study, super-resolution microscopy was considered structured illumination microscopy (SIM), and it revealed that the homodimers of the ERα and heterodimers of the ERα and ERβ isoforms were induced by estradiol (Iwabuchi *et al.* 2017*a*). SIM had a resolution of 100 nm, but we could use other devices, such as stimulated emission suppression depletion microscopy (resolution, ~60 nm) and photoactivated localization microscopy/stochastic optical reconstruction microscopy (resolution, ~20 nm) (Schermelleh *et al.* 2010). They also provided more detailed information as to the proximity of proteins examined. Furthermore, two-photon excitation (TPE) microscopy could also enable deep-tissue observation and* in vivo* imaging of living animals (Long *et al.* 2017). Adding fluorescence-lifetime imaging to TPE allows the observation of the morphology as well as intracellular signaling of deep tissue (Long *et al.* 2017). By combining FRET with two-photon fluorescence-lifetime imaging microscopy, PPIs in deep tissues can be visualized (Long *et al.* 2017).

In this review, we summarized the previously reported attempts to visualize PPIs using PLA, FRET, and super-resolution microscopy. FRET has been identified as a technique used for visualizing PPIs, including hormone-signaling-related factors. However, FRET required double transfections with separate donor- and acceptor-labeled gene constructs (Muller *et al.* 2013), which made it practically impossible to be applicable to the evaluation using FFPE tissues. In contrast, both PLA and super-resolution microscopy could be applicable to further evaluation in fixed cells and FFPE tissues (Iwabuchi *et al.* 2021*a*), whereas FRET and super-resolution microscopy to the evaluation in living cells (Schermelleh *et al.* 2010, Muller *et al.* 2013). Therefore, the analysis using those approaches could further explore the dynamics of PPIs in a more detailed fashion. On the other hand, electron microscopy (EM) has been well known to harbor a higher resolution than super-resolution microscopy (transmission electron microscopy (resolution, ~0.1 nm); scanning electron microscopy (resolution, ~0.5 nm)) (Dudkina *et al.* 2010). Therefore, EM is definitively considered an established detailed method for visualizing the localization of proteins in intracellular organelles and evaluating the proximity between the proteins. However, it is also true that the preparation of the specimens for EM is time consuming and labor intensive, including mounting and ultrathin sectioning of a sample. In addition, EM requires electron-beam irradiation under vacuum conditions. Therefore, the evaluation of biological phenomena occurring inside the living cells is difficult in the analysis employing only EM.

Both PLA and super-resolution microscopy could be applied to fixed tissues but require specific antibodies against the targeted proteins (Iwabuchi *et al.* 2021*a*). However, it is also true that both techniques could be easily implemented with a similar immunofluorescence protocol (Iwabuchi *et al.* 2021*a*). Super-resolution microscopy requires the use of expensive and specialized equipment but could allow the observation of temporal and spatial changes in intracellular organelles as well as protein localization changes and their behavior within cells. In this review, we particularly focused on PLA and FRET to further explore hormone receptor binding, hormone receptor dimerization, and hormone receptor coupling of several other proteins and their cofactors. Combining these techniques with super-resolution microscopy analysis and performing functional analysis in tissues and cells could provide pivotal information as to how these PPIs act in tissues and cells.

## Conclusions

Hormonal signaling consists of many PPIs involved in the binding of receptors to hormones, hormone receptors dimerization, forming complexes of hormone receptors and their cofactors, and forming complexes of hormone receptor and several other proteins. Visualization of PPIs will help us understand hormone signaling. Various techniques have been attempted for the visualization of hormone receptors. Super-resolution microscopy reported in this review has been used in many* in vitro* studies. Therefore, its application in tissues is expected to increase steadily in the future. In addition, evaluating the proximity of proteins using super-resolution microscopy could promote the evaluation of further fine structures. Super-resolution microscopy could evaluate FFPE tissue, which could be firstly demonstrated in routinely processed clinical materials of patients, as well as the relationship with fine structures in living cells. We believe that the application of this technology not only* in vitro* but also in actual human tissues in the future will lead to a better understanding of the pathogenesis of hormone-dependent cancers.

## Declaration of interest

The authors declare that there is no conflict of interest that could be perceived as prejudicing the impartiality of this review.

## Funding

This work did not receive any specific grant from any funding agency in the public, commercial, or not-for-profit sector.
